# Trends in mortality outcomes of hospital-admitted injury in Victoria, Australia 2001–2021

**DOI:** 10.1038/s41598-023-34114-x

**Published:** 2023-05-03

**Authors:** Janneke Berecki-Gisolf, Tharanga Fernando, Angelo D’Elia

**Affiliations:** Monash University Accident Research Centre, Monash University, Clayton Campus, Clayton, VIC 3800 Australia

**Keywords:** Health services, Public health, Epidemiology, Outcomes research

## Abstract

Due to advancements in trauma treatment methods, it is expected that survivability of hospital-admitted injuries gradually improves over time. However, measurement of trends in all-cause injury survivability is complicated by changes in case mix, demographics and hospital admission policy. The aim of this study is to determine trends in hospital-admitted injury survivability in Victoria, Australia, taking case-mix and patient demographics into account, and to explore the potential impact of changes in hospital admission practices. Injury admission records (ICD-10-AM codes S00-T75 and T79) between 1 July 2001 and 30 June 2021 were extracted from the Victorian Admitted Episodes Dataset. ICD-based Injury Severity Score (ICISS) calculated from Survival Risk Ratios for Victoria was used as an injury severity measure. Death-in-hospital was modelled as a function of financial year, adjusting for age group, sex and ICISS, as well as admission type and length of stay. There were 19,064 in-hospital deaths recorded in 2,362,991 injury-related hospital admissions in 2001/02–2020/21. Rates of in-hospital death decreased from 1.00% (866/86,998) in 2001/02 to 0.72% (1115/154,009) in 2020/21. ICISS was a good predictor of in-hospital death with an area-under-the-curve of 0.91. In-hospital death was associated with financial year (Odds Ratio 0.950 [95%CI 0.947, 0.952]), in logistic regression modelling adjusted for ICISS, age and sex. In stratified modelling, decreasing injury death trends were observed in each of the top 10 injury diagnoses (together constituting > 50% of cases). Admission type and length of stay were added to the model: these did not alter the effect of year on in-hospital death. In conclusion, a 28% reduction in rates of in-hospital deaths in Victoria was observed over the 20-year study period, in spite of aging of the injured population. This amounts to 1222 additional lives saved in 2020/21 alone. Survival Risk Ratios therefore change markedly over time. A better understanding of the drivers of positive change will help to further reduce the injury burden in Victoria.

## Introduction

Injury is a major cause of morbidity and mortality in Australia, with 13,400 injury deaths and 527,000 hospitalisations in 2019/20; this amounts to 8.4% of the burden of disease in Australia^1^. For Australians aged between one and 44 years, injury is the leading cause of death.

Trauma care systems are designed to reduce trauma related mortality. Improvements in trauma care systems are expected to lead to improvements in trauma outcomes: this has been described in trauma care systems in Canada, with improvements in risk-adjusted mortality incidence from 12.1 to 9.9% between 2006 and 2012^2^. A reduction in trauma mortality was also described in the UK, since the introduction of a national trauma care system in 2012^3^. A US study comparing mortality outcomes in level 1 trauma centres compared to hospitals without a trauma centre also reported lower risk of death in trauma centres^4^.

In Victoria, Australia, improvements in trauma outcomes have been described since the introduction of the regionalised trauma system^5,6^, both in terms of general trauma and specifically, road trauma^7^. The Victorian State Trauma Registry (VSTR), on which the analyses were based, also captures pre-hospital deaths, which is important as an increase in pre-hospital deaths could otherwise be associated with improved survival among hospital-treated trauma cases. Outside of the VSTR trauma population, improvements in hospital-treated injury outcomes in Australia have also been described for specific injury types. For example, 30-day mortality associated with hospital-treated hip fracture in a trauma centre in Newcastle, New South Wales, decreased from 12.3% in 2002 to 8.2% in 2011^8^.

It is not known if the observed improvements in mortality outcomes are limited to severe trauma, or if these trends can be observed across the full spectrum of hospital-treated injuries. In light of population ageing, such an analysis needs to take into consideration changes in patient demographics as well as injury case-mix over time. Hospital admission policy changes can affect hospital admission rates, and therefore trends in hospital admission type and setting also need to be accounted for. The aims of this study were therefore: (1) to determine time trends in injury survivability in Victoria, Australia; (2) to determine which injury types have had the greatest gains in survivability; and (3) to explore how changes in hospital admission and discharge patterns may have influenced the results.

This analysis will help identify the current strengths and weaknesses in injury patient care in Victoria, by demonstrating which patient groups have made the greatest vs. the least gains in injury survival. This will generate learnings for injury types or patient groups that are ‘left behind’ in treatment outcome progress. Furthermore, time trends in survival analysis will help to better understand how Survival Risk Ratios (SRRs) for specific injury types evolve over time. These SRR metrics are the foundation of calculation of the ICD-10 based Injury Severity Scores (ICISS)^9–11^; potential time trends in SRRs have implications for how the ICISS is generated, applied and updated.

## Methods

This is a retrospective analysis of routinely collected administrative hospital data. Admission records were analysed to determine time trends in in-hospital death (and therefore also the inverse: time trends in injury survivability). The data was further explored to quantify the impact of case-mix and hospital admission policy and practice trends: logistic regression models were constructed to model this effect.

### Data sources

The Victorian Admitted Episodes Dataset (VAED) was the source of hospital admission records for this study. All hospital admissions, public and private, in the state of Victoria, Australia, are recorded in the VAED. The recorded information includes demographic, clinical and administrative details for each hospital admitted episode of care. Forty fields capture clinical details, including injury and external cause information; these are coded according to the International Statistical Classification of Diseases and Related Health problems, 10^th^ Revision, Australian Modification (ICD-10-AM)^12,13^. The Victorian Government Department of Health supplied the VAED data to the Victorian Injury Surveillance Unit. Victorian Injury Surveillance Unit use of the VAED for injury research is approved by the Monash University Human Ethics Committee (MUHREC project number 21427).

### Case selection

Injury related hospital admissions were selected from the VAED as: all admissions that occurred between 1 July 2001 and 30 June 2021, with the first-listed ICD-10-AM diagnosis code in the range of S00-T75 or T79 (injury, poisoning and certain other consequences of external causes; complications of surgical and medical care and complications/sequelae of injuries were not included). Cases with sex coded as other than ‘male’ or ‘female’ were excluded, to prevent confidentiality concerns related to small cell counts.

### Variables

#### Sociodemographic

Age was grouped in 5-year age bands; sex was limited to male and female.

#### Injury severity

Injury severity was captured as ICISS: the ICD-10 based Injury Severity Score, using recent, Victorian diagnosis-specific survival probabilities (DSPs), based on in-hospital death^14^. Age-specific DSP of the worst injury was used. Only DSPs that were originally derived from five or more cases were used (‘conservative’ ICISS).

#### Injury type and injury group

Listings of commonly occurring specific injuries were created using the first-occurring ICD-10-AM diagnosis code, truncated at a length of three places. Injury type groups were created based on the ICD-10-AM coded injury types, and collated as: fracture; burn; open wound; crushing injury; etc.

#### Comorbidity

The Australian Injury Comorbidity Index for in-hospital death (AICI-hd) was used to capture comorbidity^15^. The following 11 conditions were included in logistic regression models: any malignancy; cardiac arrhythmias; chronic pulmonary disease; coagulopathy; congestive heart failure; dementia; metastatic solid tumour; mild liver disease; myocardial infarction; peptic ulcer disease; and renal disease including renal failure. These were included as individual binary variables and as a total sum of binary variables; because the results were similar, the latter was used as the simpler option.

#### Admission type and length of stay

Admission type was classified in several ways. First, the type of admission was categorised as emergency admission, elective admission, statistical admission (change of care type within the hospital) or other (posthumous organ procurement; maternity; birth episode). Incident admissions were also specified: admissions that were not a statistical admission or transfer; this distinction was used to tally new cases without overcounting. Finally, admissions were flagged as Emergency Department only, if they took place within the Emergency Department only, without transfer to ward. A hospital admission policy change took place in Victoria during the study period, which had a significant impact on trends in hospital admissions over time. Prior to July 2012, patients whose stay in the emergency department exceeded four hours could be recorded as a hospital admission. After July 2012, patients who received their entire care within a designated emergency department or urgent care centre could no longer be eligible for admission regardless of the amount of time spent in the hospital. Following this policy change, a decrease in hospital admissions overall was observed, followed by a relative increase in Short Stay Unit admissions^16^. Therefore, in this study, admissions that took place entirely in the Short Stay Observation Unit were also flagged, if they did not include a transfer to ward. Based on these, a composite variable was created, with the following categories: ED-only stay; Short Stay Observation Unit stay only; other stay up to one week; other stay of one week or longer.

### Statistical methods

Injury admissions per financial year and corresponding rates of in-hospital death were summarised using descriptive statistics. The recently published, Victorian ICISS were used as a static measure to account for injury severity in the case-mix, across the 20-year period. Three logistic regression approaches were used to model in-hospital death, to determine time trends in injury survivability (Model 1); to determine the effect of injury case-mix on time trends in injury survivability (Model 2); and to explore the effects of hospital admission policy and practice on measured time trends in injury survivability (Model 3). To address this, Model 1 included financial year and ICISS as continuous variables and age and sex as categorical variables; Model 2 included all variables of Model 1 plus injury type and comorbidity (AICI-hd); Model 3 included all variables of Model 1 plus admission type and stay type. Model 1 was repeated for each of the ten most commonly occurring injuries, in a stratified analysis; this was also repeated for age groups 0–14 years, 15–24 years, 25–64 years, 65–84 years and 85+ years. Firth penalised likelihood approach was used in all logistic regression models to address the issue of rare outcomes (only 1% or less of injury admissions resulted in in-hospital death).

Lives saved was calculated by applying Model 1 to the first three years of data only (2001/02 to 2003/04), and applying the parameter estimates to the 20-year sample data to calculate expected deaths in each year. For each year from 2004/05 to 2020/21, lives saved was calculated as the expected minus the observed number of in-hospital deaths. The three years (2001/02–2003/04) were deemed a sufficiently long time to generate robust parameter estimates for the relation between age, sex and the outcome; furthermore, the three years were relatively stable in terms of injury survivability.

All analysis was conducted using SAS statistical software version 9.4 (SAS Institute; Cary, North Carolina USA) and the significance was considered with the *p* value < 0.05.

### Ethics approval and consent

Victorian Injury Surveillance Unit use of the VAED for injury research is approved by the Monash University Human Research Ethics Committee (MUHREC project number 21427). The study was carried out in accordance with the specifications in the ethics application. Obtaining informed consent for this study was impracticable, due to the large number of cases: a waiver of consent was requested and provided by the Monash University Human Research Ethics Committee. The following also apply: (i) the data accessed by the researchers was de-identified; (ii) there was sufficient protection of privacy; (iii) there was an adequate plan to protect the confidentiality of the data in the research outputs.

## Results

**Figure 1:**
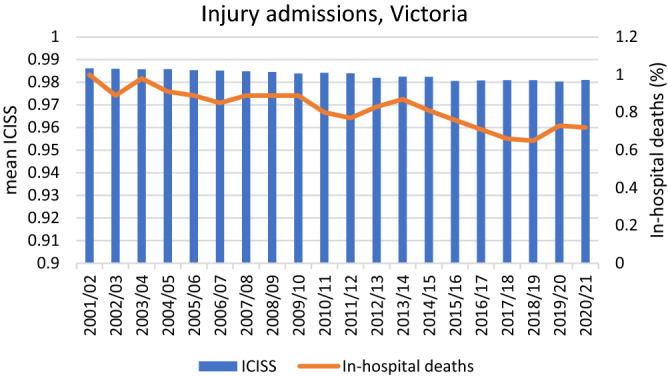
Mean ICD-based Injury Severity Score (blue bars) and percentage in-hospital deaths (orange line) in injury related hospital admissions, Victoria, 2001/02–2020/21.

In 2001/02 to 2020/21, there were 2,362,991 injury-related hospital admissions in Victoria. Of these, 309,751 were ‘ED-only’ admissions and 391,824 were ‘Short stay observation unit’ only admissions. ED only admissions mainly occurred prior to 2012, the year when the Victorian hospital admission policy change regarding ED-only admissions was implemented. Short Stay Observation Units gradually increased throughout the 20-year period, from 2% of admissions in 2001/02 to 27% of admissions in 2020/21 (Table 1). There were 19,064 in-hospital deaths: 0.77% of all injury admissions. Over the 20-year period, a 28% reduction in rates of in-hospital deaths was observed in injury admissions (Table 1, Fig. 1); a similar gradient was observed in the injury admissions that were not ED-only or short-stay unit only. Out of all 2,362,991 injury admissions, 2,015,336 (85.3%) were incident admissions. Deaths were less common in incident admissions (14,037, 0.70%) than among transfers/statistical separations (5027, 1.45%). Over the 20-year period, the injured population aged from a median of 37.6 years in 2001/02 to 53.0 years in 2020/21. The representation of older people aged 75 + years increased from 19.4% in 2001/02 to 27.4% in 2020/21. The representation of females in the injury admissions increased gradually from 43.9% in 2001/02 to 47.4% in 2020/21.

**Table 1:** Injury admissions and in-hospital deaths in Victoria, 2001/02 to 2020/21.

Financial year	Injury admissions				Death in hospital			
					Other admissions		Total	
	Short Stay Observation Unit only	Emergency Department stay only	Other admissions	Total	Deaths	Deaths/admissions (%)	Deaths	Deaths/admissions (%)
2001/02	1350	25,447	60,201	86,998	785	1.30	866	1.00
2002/03	2047	25,842	62,395	90,284	744	1.19	806	0.89
2003/04	2084	25,630	62,251	89,965	802	1.29	883	0.98
2004/05	3307	26,282	64,341	93,930	779	1.21	852	0.91
2005/06	5300	24,661	66,923	96,884	795	1.19	863	0.89
2006/07	6241	26,592	69,926	102,759	812	1.16	875	0.85
2007/08	7112	28,536	70,661	106,309	894	1.27	946	0.89
2008/09	7962	28,325	72,090	108,377	889	1.23	960	0.89
2009/10	10,756	27,347	72,262	110,365	901	1.25	979	0.89
2010/11	12,813	32,744	75,895	121,452	888	1.17	967	0.80
2011/12	14,777	32,914	79,234	126,925	901	1.14	976	0.77
2012/13	17,479	4578	79,266	101,323	836	1.05	845	0.83
2013/14	23,848	106	84,329	108,283	933	1.11	939	0.87
2014/15	28,166	118	89,937	118,221	954	1.06	959	0.81
2015/16	35,328	116	101,682	137,126	1031	1.01	1036	0.76
2016/17	40,523	135	105,733	146,391	1041	0.98	1046	0.71
2017/18	43,967	137	109,913	154,017	1015	0.92	1020	0.66
2018/19	45,787	98	112,370	158,255	1030	0.92	1034	0.65
2019/20	40,649	72	110,397	151,118	1092	0.99	1097	0.73
2020/21	42,328	71	111,610	154,009	1108	0.99	1115	0.72

During the study period, the mean ICISS was 0.983 (median 0.995; IQR 0.977 to 0.999), based on the worst recorded injury. ICISS was a good predictor of in-hospital death with an area-under-the-curve of 0.91. Over time, there was a slight gradual reduction in mean ICISS; however, during this period, there was a more pronounced reduction in the rate of in-hospital deaths (Fig. 1).

The vast majority of the admissions did not have a recorded co-morbidity that was listed in the AICI-hd (n = 2,166,207; 91.7%). Out of those that did, most had one condition (159,207; 6.7%) and only few had three or more (n = 6982; 0.3%). Of the AICI-hd listed conditions, the most commonly encountered was cardiac arrhythmias (n = 55,693; 2.4%) followed by dementia (n = 52,412, 2.2%). The prevalence of having (one or more) of these comorbidities fluctuated over time, from 6.7% in 2001/02 to a maximum of 10.3% in 2015/16, dropping to 8.4% in 2020/21.

In-hospital death was modelled as a function of financial year, adjusting for ICISS, age group and sex (Model 1, Table 2). Financial year was associated with in-hospital death with an odds ratio of 0.950 [0.947, 0.952] (parameter estimate of − 0.05 (*P* < 0.0001)). There was a strong negative association between ICISS and in-hospital death, as expected (i.e. lower survival risk ratio is expected to correspond with in-hospital death) (*P* < 0.0001). In-hospital death was positively associated with older age (*P* < 0.0001 for all age groups above 50 years, relative to 45–49 years) and male sex (*P* < 0.0001). Adding injury type and comorbidity (Model 2) did not alter the association between financial year and in-hospital death (*P* < 0.0001). Intracranial injuries, poisoning and other effects of external cause/complications/late effects (compared with fractures) were positively associated with in-hospital death (each at *P* < 0.0001). The AICI-hd comorbidity index was also positively associated with in-hospital death (*P* < 0.0001). Finally, type of admission and length of stay were introduced to the baseline model to explore the impact of hospital admission policy and practice trends on in-hospital deaths over time (Model 3). The association between financial year and in-hospital death was unaltered in this model (*P* < 0.0001) (Table 2). Compared with emergency admissions, in-hospital deaths were less common during elective admissions (*P* < 0.0001) and more common during statistical admissions (*P* < 0.0001). Hospital stay that was limited to the ED or the short stay observation unit were associated with much lower odds of in-hospital death (each at *P* < 0.0001), compared with other, longer hospital stays.

**Table 2:** Logistic regression models of in-hospital deaths, among Victorian injury admissions, 2001/02 to 2020/21. Model 1: financial year, ICD-based injury Severity Score (ICISS), age group; Model 2: Model 1 covariates and injury type group; Model 3: Model 1 covariates and hospital admission type and length of stay.

		Model 1 19,051/2,360,093	Model 2 19,051/2,360,093	Model 3 19,051/2,360,093
	N or median [range]	Odds ratio & parameter estimate*	Odds ratio & parameter estimate*	Odds ratio & parameter estimate*
Financial year*	*2012/13 [2001/02–2020/21]*	0.950 [0.947, 0.952] *− 0.05 [− 0.05, − 0.05]*	0.953 [0.950, 0.955] *− 0.05 [− 0.05, − 0.05]*	0.954 [0.951, 0.956] *− 0.05 [− 0.05, − 0.04]*
ICISS*	*0.996 [0–1.000]*	< 0.001 [< 0.001, < 0.001] *− 18.6 [− 18.9, *− *18.4]*	< 0.001 [< 0.001, < 0.001] *− 14.17 [− 14.52, − 13.82]*	< 0.001 [< 0.001, < 0.001] *− 17.39 [− 17.64, − 17.14]*
Age group				
0–4 yrs	96,496 (4.1%)	0.48 [0.38, 0.61]	0.54 [0.43, 0.68]	0.47 [0.37, 0.59]
5–9 yrs	91,252 (3.9%)	0.17 [0.12, 0.25]	0.18 [0.13, 0.26]	0.16 [0.11, 0.22]
10–14 yrs	99,905 (4.2%)	0.18 [0.13, 0.25]	0.18 [0.13, 0.26]	0.17 [0.12, 0.24]
15–19 yrs	158,520 (6.7%)	0.45 [0.38, 0.54]	0.47 [0.39, 0.57]	0.46 [0.38, 0.55]
20–24 yrs	176,823 (7.5%)	0.53 [0.44, 0.62]	0.56 [0.48, 0.67]	0.55 [0.46, 0.65]
25–29 yrs	152,173 (6.4%)	0.75 [0.64, 0.89]	0.79 [0.67, 0.93]	0.79 [0.67, 0.93]
30–34 yrs	133,527 (5.7%)	0.72 [0.60, 0.85]	0.75 [0.63, 0.89]	0.75 [0.63, 0.89]
35–39 yrs	123,622 (5.2%)	0.87 [0.73, 1.03]	0.89 [0.75, 1.05]	0.89 [0.75, 1.05]
40–44 yrs	119,360 (5.1%)	0.95 [0.81, 1.13]	0.97 [0.82, 1.14]	0.96 [0.81, 1.13]
45–49 yrs (ref)	115,655 (4.9%)			
50–54 yrs	109,421 (4.6%)	1.31 [1.12, 1.54]	1.27 [1.09, 1.49]	1.29 [1.10, 1.51]
55–59 yrs	104,655 (4.4%)	1.47 [1.25, 1.72]	1.35 [1.15, 1.59]	1.42 [1.21, 1.66]
60–64 yrs	99,022 (4.2%)	1.98 [1.70, 2.30]	1.75 [1.50, 2.04]	1.89 [1.63, 2.20]
65–69 yrs	96,866 (4.1%)	1.81 [1.57, 2.09]	1.57 [1.35, 1.81]	1.74 [1.51, 2.01]
70–74 yrs	106,678 (4.5%)	2.82 [2.47, 3.23]	2.17 [1.89, 2.48]	2.70 [2.36, 3.09]
75–79 yrs	128,178 (5.4%)	4.11 [3.62, 4.67]	2.90 [2.54, 3.30]	3.91 [3.44, 4.45]
80–84 yrs	159,516 (6.8%)	2.88 [2.54, 3.26]	2.21 [1.95, 2.52]	2.84 [2.50, 3.22]
85 + yrs	291,322 (12.3%)	5.42 [4.80, 6.13]	4.04 [3.56, 4.57]	5.36 [4.74, 6.06]
Sex				
Male (ref)	1,278,791 (54.1%)			
Female	1,084,200 (45.9%)	0.66 [0.64, 0.68]	0.71 [0.68, 0.73]	0.66 [0.64, 0.68]
Injury type				
Fracture (ref)	978,629 (41.4%)			
Burns	31,106 (1.3%)		1.12 [0.96, 1.30]	
Crushing injury	4708 (0.2%)		0.46 [0.20, 1.08]	
Dislocation, sprain & strain	143,763 (6.1%)		0.28 [0.24, 0.33]	
Eye injury- excl foreign body	11,230 (0.5%)		0.33 [0.21, 0.52]	
Foreign body	38,558 (1.6%)		1.18 [1.03, 1.37]	
Injury to blood vessels	15,900 (0.7%)		1.64 [1.33, 2.02]	
Injury to internal organs	25,784 (1.1%)		1.57 [1.44, 1.76]	
Injury to muscle & tendon	98,048 (4.2%)		0.14 [0.10, 0.18]	
Injury to nerves & spinal cord	27,570 (1.2%)		1.12 [0.97, 1.30]	
Intracranial injury	102,836 (4.4%)		1.85 [1.76, 1.95]	
Open wound	324,854 (13.8%)		0.32 [0.29, 0.35]	
Other and unspecified injury	213,748 (9.1%)		0.50 [0.46, 0.55]	
Other effects of ext cause/complications/late effects	18,131 (0.8%)		2.89 [2.64, 3.15]	
Superficial injury	123,354 (5.2%)		0.43 [0.39, 0.48]	
Systemic-poisoning/toxic effects	182,020 (7.7%)		1.39 [1.28, 1.49]	
Traumatic amputation	22,752 (1.0%)		0.12 [0.06, 0.24]	
Australian Injury Comorbidity index- hospital death*	*0 [0–7]*		2.44 [2.40, 2.48] *0.89 [0.87, 0.91]*	
Admission type				
Emergency† (ref)	1,749,429 (74.0%)			
Elective admission	577,456 (24.4%)			0.47 [0.45, 0.49]
Statistical admission	33,577 (1.4%)			1.23 [1.15, 1.31]
Other‡	2529 (0.1%)			0.14 [0.02, 1.14]
Stay				
Emergency Department only	309,751 (13.1%)			0.26 [0.24, 0.28]
Short Stay Observation Unit only	391,824 (16.6%)			0.02 [0.02, 0.03]
Other stay up to 1 week (ref)	1,294,898 (54.8%)			
Stay one week or longer	366,518 (15.5%)			0.83 [0.80, 0.86]

*For continuous variables (*in italics*), results are shown as parameter estimates as well as odds ratios.

^†^Emergency admission through the hospital’s ED and other emergency admissions.

^‡^Posthumous organ procurement/Maternity/Birth episode.

In order to better understand the downward trend in in-hospital death rate and whether this was true for all injury admissions or limited to subgroups, such as specific injury types or demographics, stratified models were applied (Table 3). Selecting the ten most commonly occurring specific injuries, which together constituted 50% of all injury admissions, application of Model 1 (financial year, age group, sex) showed that the downward trend in in-hospital deaths was observed across all ten injury types. The reduction was most pronounced in admissions for wrist and hand fractures (parameter estimate: − 0.073) and least pronounced in admissions for forearm fractures (parameter estimate: − 0.036). Notably, in-hospital death was very rare in some injury types such as fracture at wrist and hand level and open wound of wrist and hand. Even without adjustment for age, improvements in survival can be demonstrated in the raw data, for specific injuries. For example in 2001/02, 326 of the 6474 hip fracture admissions resulted in in-hospital death (5.0%); in 2005/6, 307/6704 died (4.6%); in 2010/11, 304/7560 died (4.0%); in 2015/16, 315/11,470 died (2.8%) and in 2020/21, 309/10,876 died (2.8%). Reductions in in-hospital death over time were also demonstrated for severe injuries (ICISS ≤ 0.941) and non-severe injuries (ICISS > 0.941) (Table 3). Negative associations between financial year and the outcome, in-hospital death, were observed in all five age groups: most so for the ages 0–14 years (OR 0.939 [0.913, 0.965], parameter estimate: − 0.063) and least so for the ages 25–64 years (OR 0.982 [0.975, 0.989], parameter estimate: − 0.018).

**Table 3:** Stratified analysis of injury fatality trends over time, for the ten most common injury diagnoses (coded to ICD-10-AM) and age in five major groups.

		Logistic regression model of in-hospital death*	
Ten most common injury diagnoses: ICD-10-AM†	In-hospital deaths/admissions N (%)	Financial year: Odds ratio [95% CI] and model parameter estimate [95% CI], *p* value	
Fracture of forearm (S52)	154/174,524 (0.09%)	0.965 [0.939, 0.991] − 0.036 [− 0.063, − 0.009]	*P* = 0.009
Fracture of femur (S72)	6069/170,050 (3.57%)	0.955 [0.951, 0.959] − 0.046 [− 0.051, − 0.042]	*P* < 0.0001
Fracture of lower leg, including ankle (S82)	267/141,340 (0.19%)	0.956 [0.936, 0.976] − 0.045 [− 0.066, − 0.024]	*P* < 0.0001
Open wound of head (S01)	242/128,409 (0.19%)	0.950 [0.930, 0.971] − 0.051 [− 0.073, − 0.030]	*P* < 0.0001
Fracture at wrist and hand level (S62)	21/111,547 (0.02%)	0.930 [0.873, 0.991] − 0.073 [− 0.136, − 0.009]	*P* = 0.02
Fracture of shoulder and upper arm (S42)	615/106,548 (0.58%)	0.944 [0.931, 0.957] − 0.058 [− 0.072, − 0.044]	*P* < 0.0001
Intracranial injury (S06)	4726/102,836 (4.60%)	0.962 [0.957, 0.968] − 0.039 [− 0.044, − 0.033]	*P* < 0.0001
Open wound of wrist and hand (S61)	21/96,424 (0.02%)	0.933 [0.877, 0.992] − 0.070 [− 0.131, − 0.008]	*P* = 0.03
Fracture of skull and facial bones (S02)	222/80,585 (0.28%)	0.944 [0.922, 0.966] − 0.058 [− 0.081, − 0.034]	*P* < 0.0001
Fracture of rib(s), sternum and thoracic spine (S22)	923/73,447 (1.26%)	0.950 [0.940, 0.961] − 0.051 [− 0.062, − 0.039]	*P* < 0.0001
ICISS			
≤ 0.941 severe injury	12,658/204,195 (6.20%)	0.955 [0.952, 0.958] − 0.046 [− 0.049, − 0.042]	*P* < 0.0001
> 0.941 non severe injury	6393/2,143,240 (0.30%)	0.950 [0.946, 0.954] − 0.052 [− 0.056, − 0.048]	*P* < 0.0001
Age in major groups			
0–14 years	175/286,706 (0.06%)	0.939 [0.913, 0.965] − 0.063 [− 0.091, − 0.036]	*P* < 0.0001
15–24 years	471/334,890 (0.14%)	0.969 [0.952, 0.986] − 0.032 [− 0.049, − 0.014]	*P* = 0.0005
25–64 years	2524/956,934 (0.26%)	0.982 [0.975, 0.989] − 0.018 [− 0.026, − 0.011]	*P* < 0.0001
65–84 years	6520/490,491 (1.33%)	0.947 [0.943, 0.951] − 0.054 [− 0.059, − 0.050]	*P* < 0.0001
85 + years	9361/291,072 (3.22%)	0.950 [0.947, 0.954] − 0.051 [− 0.055, − 0.047]	*P* < 0.0001

*Adjusted for age, sex, ICISS.

^†^10 most common (principal) diagnoses make up 50% of the injury cases.

**Figure 2:**
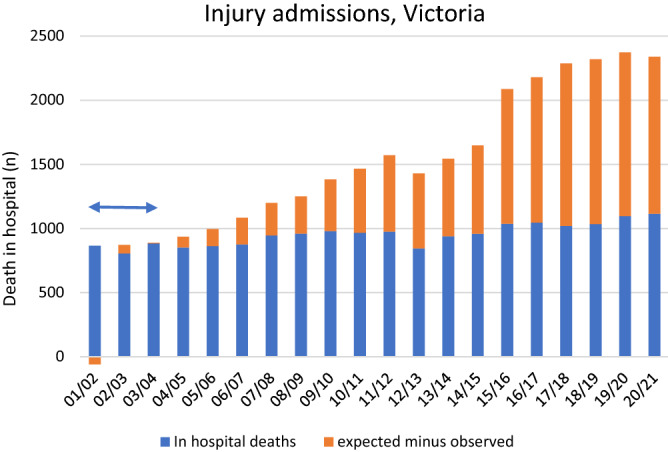
Injury admission in-hospital deaths in Victoria, 2001/02 to 2020/21. Observed cases across the 20-year period, and observed minus expected cases (based on logistic regression modelling of 2001/02–2003/04). Blue arrow indicates the period that was used to generate parameter estimates for calculating expected cases.

Estimates of lives saved due to improved survivability of hospital-treated injury in Victoria were generated by applying the Model 1 parameter estimates generated from the first three years of data (2001/02–2003/04) to the full 20 years of injury admission records and calculating the resulting proportions. The number of observed in-hospital deaths was subtracted from the number of in-hospital deaths expected based on the modelled first three years. The results are shown in Fig. 2: over the 17-year period from 2004/05 to 2020/21, it is expected that 11,574 deaths were prevented due to improved survivability of hospital admitted injury. In 2020/21 alone, an estimated 1223 deaths were prevented, when considering 2001/02–2003/04 injury survivability.

## Discussion

In this study, a retrospective hospital admissions data analysis was conducted to determine time trends in hospital-admitted injury survival over a twenty-year period in Victoria, Australia. It was found that hospital-admitted injury survival improved statistically significantly, and this effect was unchanged after accounting for changes in case-mix and hospital admission policy changes over time. The improvement in survival was observed across each of the ten most commonly encountered injury types, all broad age groups, and it was not limited to severe injuries.

Improved injury survival has been reported for trauma patients since the introduction of the regionalised trauma system in Victoria^5–7^; notably, improved survival did not result in increased disability but in fact coincided with improved functional outcomes at twelve months. The observed improvements in survival could potentially be related to treatment at major trauma services (i.e., level-1 trauma centres), clinical experience in such centres, and coordinated care pathways^7^. In the current study of outcomes of hospital admitted injury in Victoria, improved survival was not limited to severe injuries: in fact, improvements were slightly greater among non-severe injuries. Any explanation of why injury patient survival has improved over the twenty-year period is speculative. Statistical adjustment for changes in case-mix, demographics, admission type and comorbidity rule out the most direct explanations. Potentially, the observed improved survival could be related to improved clinical care over time. With the constant advancements in medical technology and clinical practice improvements, this is plausible.

This study did not investigate the cause of death. While in-hospital deaths were the recorded end-point of an injury-related hospital admission, non-injury conditions may have contributed to the death. Although comorbidity was adjusted for in statistical modelling, not all comorbidities are recorded in hospital data, and severity and management of comorbidity are not captured. In Australia, there has been a drastic reduction in ischaemic heart disease deaths between 1968 and 2017, from 428 to 59 deaths per 100,000 population, respectively^17^. This is generally attributed to improved medical interventions, medications and health promotion campaigns^17,18^. Potentially, underlying improvements in cardiovascular health in the Australian population over the 20-year study period may have contributed to improved survival of injury patients. Further research involving detailed chart review of comorbid conditions, or linkage of hospital records to death data to determine underlying and contributing causes of death, would be useful to determine drivers of the improved survival trends.

A notable increase in injury patient age was observed in this study of Victorian hospital admitted injury patients, over the 20-year period from 2001/2 to 2020/21. This could be related to population ageing: in Australia, the proportion of the population that was aged 75 years and above increased from 5.6% in 2000 to 7.1% in 2022^19^. Specifically, the proportion of those aged 85 years and above increased from 1.3 to 2.1% during this period^19^. Although this proportion is small overall, the change over time constitutes a 56% increase, in the age group with the highest rates of injury hospitalisations, i.e. the frailest group. A more general ageing of hospitalised patients (not limited to injury patients) has been reported by others: in 2004/5 to 2013/14, the proportion of hospital-admitted patients aged 85 years and above increased by 7.0% per year in Australia^20^. Although a change in hospital practices may have contributed to the pattern observed in this study, the incremental year-by-year increase in the age of the injured population over time suggests a gradual trend. Notably, increased injury survivability was observed in this study *in spite of hospital patient population ageing* over time; all statistical models were subsequently age-adjusted to account for demographic changes. Future research focussed on further exploration of age trends in hospital-admitted injury is warranted to better understand these demographic shifts in admissions.

Change in rate of in-hospital death over time has implications for calculation of the ICISS. If injury-specific survival risk ratios (SRRs) change over time, this means that SRRs derived from earlier periods result in underestimates of expected survival, when applied to more recent data. In a recently derived ICISS measure for Victoria, serious injury rates, determined by a cut-off value of 0.941^21,22^, were much lower using the new measures for in-hospital death than using older, published Australian values^11,14^. In light of the observed improved survival of hospital-treated injury over the past 20 years, an amendment of the cut-off value of 0.941 should be considered. With injury in-hospital death rates of 0.72% in Victoria in 2020/21, recent and locally derived SRRs will identify only very few cases as ‘serious injury’, using the cut-off based on 6% mortality. In this setting, ICISS should be considered as a relative measure of serious injury only, not as a realistic estimate of threat to life. Older SRRs applied to new data will render *low specificity*, whereas new SRRs applied to older data will render *low sensitivity*. Furthermore, the current, low in-hospital mortality rates suggest that high threat to life, calculated using survival risk ratios*,* may no longer be the best way to classify serious injury. Other markers that are available in administrative hospital data, such as ICU stay and total number of days in hospital, could be considered as alternative outcomes.

## Study strengths and limitations

The strength of this study is its population-based approach using hospital admission data from an entire State in Australia. This allowed for comprehensive modelling of in-hospital death, with a range of statistical adjustments to strengthen the reliability of the findings. There are, however, study limitations that need to be acknowledged. First, this study is limited to hospital treated injury. Pre-hospital deaths, i.e. injury cases that died without medical treatment are not included. It is therefore possible that improvements in survival in hospital coincided with increased *pre-hospital* death. However, morbidity trends during this period do not suggest an overall increase in injury deaths: Australia-wide, between 2010–2011 and 2019–2020 there was an average annual *decrease* in the rate of injury death of 0.1%^23^. Second, the data used in this study was unlinked: all injury-related hospital admission records were included, but these were not grouped into periods of care. Analysis of periods of care will result in higher rates of in-hospital mortality, as the numerator stays the same (in-hospital deaths) but the denominator decreases (periods of care vs. all admissions). The effects of statistical admissions (changes in care type within the hospital) have been accounted for in the modelling (i.e., model 3). A data linkage study would be useful to determine the impact of health history (including a comprehensive set of comorbidities) on injury mortality, and to determine post-discharge mortality, such as death within 30 days. Analysis of 30-day mortality, using linked data, would help to identify more subtle risk factors and trends in injury outcomes. Furthermore, analysis of linked data would allow for repeated measures modelling: one person could be admitted to hospital for an injury more than once during the study period. Repeated measures modelling takes this effect into account. However, given: (i) the effect size and level of statistical significance in the current results; (ii) the fact that *repeat injury admissions* are unlikely to be a common occurrence and (iii) the outcome in the current study is cross sectional (i.e. recorded in the same hospital admission record, not followed over time), repeated measures modelling is unlikely to change the overall findings. Third, this study was limited to in-hospital death and functional outcomes or quality of life were not considered. Ideally, improved clinical care would result in greater survival as well as improved functional outcomes, rather than improved survival at the cost of functional outcomes. This was not evaluated in the current study: future studies incorporating follow-up of patients (for example, through surveys) could address this.

In conclusion, the results of this retrospective administrative hospital data study demonstrate improved survival of hospital-treated injury in Victoria over a twenty-year period. Improved patient care, advances in medical technology, and improvements in the underlying health of the population in terms of cardiovascular health and management of chronic conditions, could potentially all have contributed to this; further studies using a data linkage approach could clarify help to determine contributing factors and areas for further improvement. This study demonstrates changes in injury survivability over time, and it is therefore recommended that this effect is considered when applying survival risk ratios to determine injury severity. Older survival risk ratios applied to new hospital-treated injury data will not render a realistic estimate of high threat to life.

## Data Availability

The data that support the findings of this study are available from the Victorian Agency for Health Information (VAHI), Victorian Department of Health, but restrictions apply to the availability of these data, which were used under Victorian Injury Surveillance Unit (VISU) approval for the current study, and so are not publicly available. Data are, however, available from the data custodians (VAHI) on request (pending ethical and data custodian approvals). Aggregate (non unit-record) injury data can be requested directly from VISU.
